# Comparison between Subsequent Irradiation and Co-Irradiation into SIMP Steel

**DOI:** 10.3390/ma14061393

**Published:** 2021-03-12

**Authors:** Yong Wang, Tongmin Zhang, Qing Liao, Junyuan Yang, Weigang Gu, Yongfei Ren, Zheng Jia, Bingsheng Li

**Affiliations:** 1 China Institute for Radiation Protection, Taiyuan 030006, China; cirpguweigang@163.com (W.G.); renyongfei0603@163.com (Y.R.); Jz15383433932@163.com (Z.J.); 2 Institute of Modern Physics, Chinese Academy of Sciences, Lanzhou 730000, China; ztm@impcas.ac.cn; 3 State Key Laboratory for Environment-Friendly Energy Materials, Southwest University of Science and Technology, Mianyang 621010, China; liaoqing@163.com (Q.L.); yangjy2019@sina.com (J.Y.); 4 Engineering Research Center of Biomass Materials, Ministry of Education, Southwest University of Science and Technology, Mianyang 621010, China

**Keywords:** martensitic steel, Fe and He irradiation, microstructure

## Abstract

A modern Chinese ferritic/martensitic steel SIMP, is a new perspective nuclear structural material for the spallation target in accelerator driven sub-critical system. In this work, aimed at exploring the radiation resistance properties of this material, we investigate the differences between simultaneous Fe and He ions irradiation and He implantation of SIMP steel pre-irradiated by Fe self-ions. The irradiations were performed at 300 °C. The radiation-induced hardening was evaluated by nano-indentation, while the lattice disorder was investigated by transmission electron microscopy. Clear differences were found in the material microstructure after the two kinds of the ion irradiation performed. Helium cavities were observed in the co-irradiated SIMP steel, but not the case of He implantation with Fe pre-irradiation. In the same time, the size and density of Frank loops were different in the two different irradiation conditions. The reason for the different observed lattice disorders is discussed.

## 1. Introduction

Nuclear energy is a leading source of low-carbon electricity in the world. Together with renewables, it provides an essential role in the global energy transition. However, three main concerns to be considered: (1) ensuring a sustainable and responsible use of resources; (2) minimizing the probability and consequences of major nuclear accidents; (3) reducing the production of long-lived radionuclides, e.g., ^129^I, ^137^Cs, ^90^Sr, ^99^Tc in nuclear waste. In order to address all three aspects, the concept of an accelerator driven sub-critical (ADS) system was proposed in the 1990s, containing spallation neutron source, which enables usf to run a subcritical nuclear reactor and to use an existing spent fuel from PWR reactors. In particular, the decay time of the long lived radionuclide can be reduced by a factor 1000 to time scales that are below 1000 years, resulting in shorter-term hazard and less heat from the waste. The Chinese Academy of Sciences (CAS) performed a strategic project of advanced nuclear fission energy in the future since 2011 [1]. Using a very high power of neutron transmutes the long-lived radionuclide into short-lived medium elements, as well as from less than 1% of the fuel’s energy content in light water reactors to above 95% in ADS. Due to its a low melting point, low vapor pressure, high atomic number and high thermal conductivity, lead-bismuth eutectic (LBE) is considered the primary candidate material used in spallation target and coolant. One of the serious problems using LBE is that containment materials, such as ferritic-martensitic (FM) steels, suffer from LBE corrosion [2,3]. Oxidation, dissolved corrosion and liquid metal embrittlement possible occur when FM steels contact with LBE. In order to lower the influence of LBE corrosion, a new type of reduced activation martensitic steel, SIMP steel (Fe-10.7 Cr–1.4 Si–1.2 W–0.5 Mn–0.2 C–0.2 V, wt.%) with 1.4 wt.% Si, was corporately developed by the Institute of Modern Physics, CAS and Institute of Metal Research, CAS. In ADS operation surroundings, SIMP steel suffers from synergistic influence of neutron irradiation, LBE corrosion and high temperature. Therefore, it is vital to assess the property changes of SIMP steel under particle irradiation and LBE corrosion.

The use of ion irradiation as a surrogate for neutron irradiation is a convenient way to assess the radiation resistance of nuclear material, in a reasonable time and without any induced radioactivity. So far, most of reports of ions irradiated SIMP steel using single-beam ions [4,5,6,7]. It is well known that a great number of helium atoms are formed by the (n, α) nuclear reaction. We can use Fe ion irradiation to simulate neutron irradiation-induced lattice displacement. The production of transmutation helium in the material can be effectively simulated by He ion implantation [8,9,10]. Due to the simultaneous occurrence of lattice disorder and helium accumulation, simultaneous dual-beam implantation of Fe and He seems to be an optimal procedure for an experimental simulation of realistic radiation condition. However, there are many reports published in the literature where subsequent irradiations are performed, either with pre-implanted Fe followed by He implantation or with pre-implanted He followed by Fe implantation [11,12,13]. Wei et al. [11] reported irradiation hardening under He and subsequent Fe ions irradiation in CLAM steel (Fe–8.9 Cr–1.5 W–0.5 Mn–0.2 V–0.1 C, wt.%) at RT. They argued significant irradiation hardening via He ions irradiation to fluences of 0.7–1.0 × 10^17^ ions/cm^2^ at room temperature (RT), and less contribution of the subsequent Fe ions irradiation to a fluence of 5.0 × 10^15^ ions/cm^2^ at RT. It is still an open question whether subsequent irradiation can simulate co-irradiation, which needs elucidate what difference between subsequent irradiation and simultaneous irradiation. In this study, we investigated the microstructure of SIMP steel irradiated by Fe + He simultaneously and Fe + He subsequently at 300 °C. The irradiation temperature is within the operation surroundings of ADS and pressurized water reactor. Dislocation loops are particularly concerned, because clusters of self-interstitial atoms and interstitial-type dislocation loops are of the main microstructural damage features at intermediate temperatures (room temperature to 300 °C). These defects act as obstacles for dislocation movement, resulting in irradiation hardening and the increase in ductile-to-brittle transition temperature (DBTT).

## 2. Experimental Process

**Figure 1:**
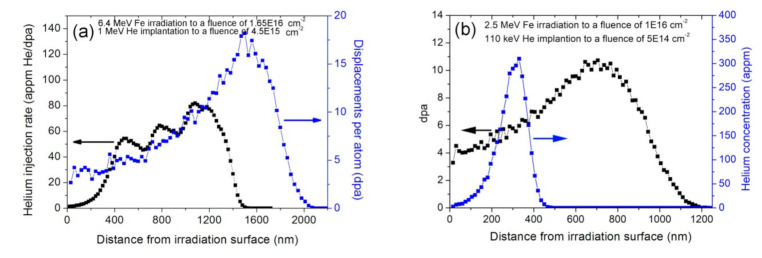
Depth profiles of displacement damage induced by Fe irradiation and of helium deposition via (**a**) dual-beam, (**b**) single-beam, simulated by SRIM-2008.

The composition of SIMP steel is given in Table 1. The sample surface was polished by sandpaper down to 4000 grit and then mirror-like polished with 0.1 diamond spray. The sample dimension was 5 × 3 × 1 mm^3^. Dual-beam irradiation experiment was carried out at the dual-Beam Material Irradiation Facility for Energy Technology (DuET) at the Institute of Advanced Energy, Kyoto University. The experiment facility was described in Ref. [14]. The energy of Fe^3+^ ions was 6.4 MeV provided by a 1.7 MeV tandem accelerator. The energy of He^+^ ions was 1 MeV supplied by a 1 MV Singletron accelerator. In order to provide a uniform distribution of helium atoms in a certain depth range, the helium beams went through three different thicknesses of alumina films. The Fe and He fluences were 1.65 × 10^16^ ions·cm^−2^ and 4.5 × 10^15^ ions·cm^−2^, respectively. The irradiation temperature was well controlled at 300 ± 5 ℃ detected by an infrared thermometer. The Monte-Carlo code Stopping and Range of Ions in Matter (SRIM-2008) quick cascade simulations was used to simulate depth profiles of the displacement damage induced by 6.4 MeV Fe^3+^ irradiation and the helium deposition (using the threshold displacement energy of 40 eV and the density of 7.86 g·cm^−3^) [9]. The simulated result is illustrated in Figure 1a. The nominal displacement damage rate, nominal displacement damage were 3 × 10^−4^ dpa·s^−1^ and 5 dpa at a depth of 600 nm, respectively. The helium injection ratio was 60 appm He.dpa^−1^ in the corresponding depth. Single-beam irradiation experiment was carried out at 320 kV High-voltage Platform in the Institute of Modern Physics, Chinese Academy of Sciences. 2.5 MeV Fe^10+^ ions to a fluence of 1 × 10^16^ ions·cm^−2^ at 300 °C irradiated SIMP steel, and then 110 keV He^+^ ions to a fluence of 5 × 10^14^ ions·cm^−2^ at 300 °C implanted the Fe pre- irradiated sample. Figure 1b shows that the displacement damage and helium injection ratio were 5 dpa and 60 appm He dpa^−1^ at a depth of 300 nm, respectively; therefore, it gets the same displacement damage and helium concentration as the Fe and He co-irradiation. Note that the displacement contribution by He implantation was only 0.01 dpa, which is far smaller than the elastic collision induced by Fe irradiation. The Fe^10+^ flux was kept at 6 × 10^11^ cm^−2^ s^−1^ and the He^+^ flux was kept at 3 × 10^12^ cm^−2^ s^−1^. The depth profiles of the displacement damage induced by 2.5 MeV Fe^10+^ irradiation and the helium deposition were simulated by SRIM-2008 [15], as shown in Figure 1b.

Nano Indenter G200 with a Berkovich-type indentation tip with a load resolution of 50 nN and displacement resolution of 0.01 nm was used to measure the nano-indentation hardness. The continuous stiffness measurement (CSM) was carried out to characterize the hardness-depth relationship. The maximum penetration depth was 1.2 μm with a strain 0.05 s^−1^ rate. A harmonic displacement of 2 nm, 45 Hz frequency and Poissons Ratio of 0.3 at room temperature, was set. A fused silica reference specimen was used to calibrate the indenter tip geometry. The Oliver–Pharr method was performed to calculate the hardness change. For each sample, fifteen testing points with 50 μm interval were set and the averaged hardness was calculated based on the measured hardness data. Post irradiation, the sample was characterized by a JEOL 2010 TEM operated at 200 kV. Cavities were observed via Fresnel contrast, where they exhibit bright Fresnel fringes in under-focused condition, but dark Fresnel fringes in over-focused condition [10]. Lattice disorder was observed by dynamical two-beam bright-field, as well as weak-beam dark-field diffraction conditions. Cross-sectional TEM (XTEM) samples were fabricated to investigate the depth distribution of radiation-induced damage. XTEM samples were obtained by Hitachi 2000 focused ion beam (FIB) system. Initially 30 keV was used and finally 5 keV was used. Electrochemical polishing for 15 ms at −45 ℃ was performed to remove the Ga ions-induced damage during FIB fabrication. Initially deposited a 1–2 μm thickness tungsten film on the irradiated surface, TEM-lamella sample was extracted by 30 kV Ga ions. The sample thickness was measured by convergent beam electron diffraction (CBED) method. The size and number density of the observed cavities were measured by Nano Measurer software with an uncertainty of approximately ±20% [16].

## 3. Results and Discussion

**Figure 2:**
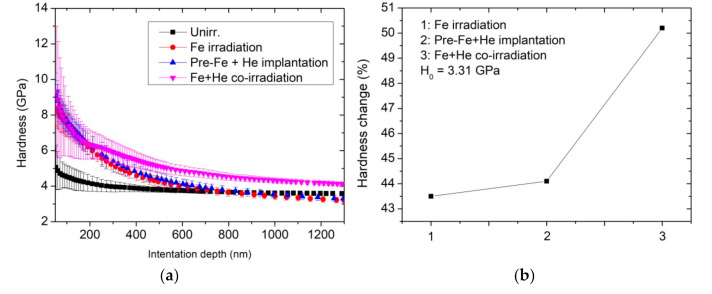
(**a**) Indentation-depth dependence of the nano-indentation hardness of samples after Fe irradiation, Fe pre-irradiation + He implantation and Fe + He co-irradiation, compared to the as-irradiated sample, (**b**) the hardness change (%) after three different irradiation conditions.

Figure 2 presents the hardness profile with indentation depth. It can be seen that the hardness value of the unirradiated sample decreases monotonously with increasing indentation depth. It can be accounted for the indentation size effect, which is often observed in crystalline materials and related to the increasing contribution of geometrically necessary dislocations at small scales [17]. Similarly, the hardness values of samples after Fe irradiation and Fe pre-irradiation + He implantation also decrease monotonously with indentation depth. The difference is that the hardness value at depths ranging from 50–700 nm is significantly larger than that of the unirradiated sample. The hardness values between the Fe irradiation and Fe pre-irradiation + He implantation have little difference, which indicates that the He implantation does not introduce pronounce hardness. However, the Fe and He co-irradiated sample has different curve change. There is an inflection at a depth of approximately 300 nm, which is about one fifth of the Fe ion projected range. According to the plastic deformed theory, the depth of the deformed region beneath the indent was approximately 4–5 times indentation depth [18]. This result demonstrates there is an obvious hardening layer inside the sample. Using Nix-Gao model and Kasada approach [19,20] the hardness value in the unirradiated sample is 3.31 GPa, and hardness increased about 43.5%, 44.1–4.75% GPa, 4.77 GPa after Fe irradiation and Fe pre-irradiation + He implantation, respectively. However, the hardness change obviously increases about 50.2–4.97% GPa after Fe and He co-irradiation. Therefore, the Fe and He co-irradiation can enhance hardness of the damaged layer. Lee et al. [21] investigated triple ion beams studies of radiation damage in 9 Cr–2 WVTa at 80, 200 and 350 °C, and reported the severe hardening in Fe + He + H, followed by Fe + He, Fe, Fe + H, He and He + H beams. The present result is consistent with Lee’s report. According to the Orowon’s theory for athermal bowing of dislocations around obstacles on a slip plane [22], the hardening is proportional to square root of the number density and diameter of obstacles, such as irradiation-induced dislocation loops, cavities and precipitates. Therefore, it is necessary to observe microstructure change in the different irradiated samples.

**Figure 3:**
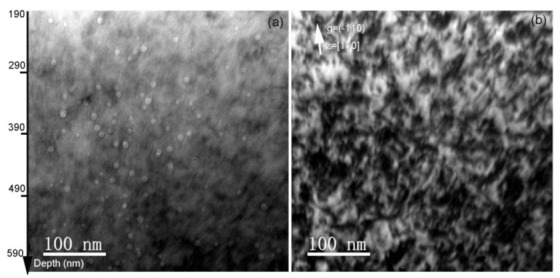
(**a**) Bright-field XTEM micrograph of irradiation-induced dislocation loops in the Fe and He co-irradiated SIMP steel to fluences of 1.65 × 10^16^ Fe^3+^·cm^−2^ and 4.5 × 10^15^ He^+^·cm^−2^. Micrographs were taken near [110] zone axis under the kinematic condition, ***g*** = (−110), (**b**) Bright-field TEM micrograph of cavities formed in the Fe and He co-irradiated SIMP steel. The under-focused view with Δ**f** = −512 nm was performed to characterize cavity distribution.

Figure 3 shows the depth distribution from 200 to 700 nm of the observed lattice defects and cavities formed by the Fe and He co-irradiation, where helium deposition was calculated by SRIM-2008. In bright-filed condition, a great number of defects, including black spots, ellipse-shaped loops and lines, are exhibited black contrasts. In general, black spots are regarded as the initial growth stage of ellipse-shaped loops; thus, black spots and ellipse-shaped loops belong to dislocation loops [23]. The size and density of observed dislocation loops are analyzed. In addition, many cavities observed under the under-focused condition, and the distribution zone is consistent with helium deposition simulated by SRIM-2008. No preferential nucleation of cavities along grain boundaries or dislocations is observed. It is indicated that the migration rate of irradiation-induced vacancies is limited at 300 °C, rather than the case of 400 °C and above irradiation that cavities are inclined to form along grain boundaries and dislocations, resulting in increasing risk of brittle-fracture [24].

**Figure 4:**
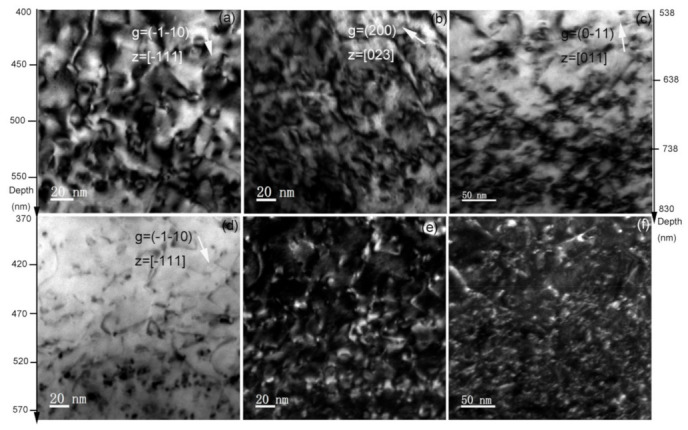
TEM micrographs of irradiation-induced dislocation loops in the He implantation with Fe pre-irradiation into SIMP steel to fluences of 1 × 10^16^ Fe^10+^·cm^−2^ and 5 × 10^14^ He^+^·cm^−2^, under bright-field (**a**–**d**), and weak-beam dark-field (**e**). In comparison, the microstructures observed by bright-field and weak-beam dark field in the Fe-irradiated sample to a fluence of 1 × 10^16^ Fe^10+^·cm^−2^ were presented in (**c**–**f**), respectively.

Figure 4 shows the depth distribution from 350 to 700 nm of lattice defects, including dislocation loops and dislocation lines in the He implantation with Fe pre-irradiation into SIMP steel. The dislocation loops were formed by Fe irradiation and dislocation lines exist in the as-grown sample. The lattice defects can be observed by the two-beam and weak-beam dark-field conditions. In the two-beam bright-field condition, dislocation loops were easily observed due to a strong diffraction contrast. Figure 4a,b shows the dislocation loops observed by the two-beam condition with diffraction factors, ***g*** = (−1–10) and ***g*** = (200), respectively. It can be seen that the number of the observed dislocation loops observed by ***g*** = (200) is approximately 3.01 × 10^22^ m^−3^, while it is approximately 2.23 × 10^22^ m^−3^ with ***g*** = (−1–10). The number density of the observed dislocation loops was obtained by the following relationships based on ***g·b*** contrast and assuming only 1/2 <111> and <100> loops exist. d_111_ + 1/3d_100_ = A for ***g*** = (200), and 1/2d_111_ + 2/3d_100_ = B for ***g*** = (110), where d_111_ and d_100_ are the number density of 1/2 <111> loops and <100> loops, respectively. A and B are the number density of the visible loops with ***g*** = 200 and ***g*** = 110, respectively. Hence, it can get d_111_ = 2.52 × 10^22^ m^−3^ and d_100_ = 1.45 × 10^22^ m^−3^. It demonstrates that the most of the observed dislocation loops have Burgers vectors ***b*** = 1/2<111>, which is consistent with some reports that 1/2<111> loops were easily formed at 300 °C and below [25,26]. Figure 4d,e shows the lattice defects observed by the weak-beam bright-field and weak-beam dark-field conditions, respectively. Some ellipse-shaped defects and many black spots were observed. The size of the observed lattice defects was evidently smaller than that observed by the two-beam condition, while the number of the observed lattice defects was larger than that observed by the two-beam condition. It should be noted that no cavities were observed in this region.

**Figure 5:**
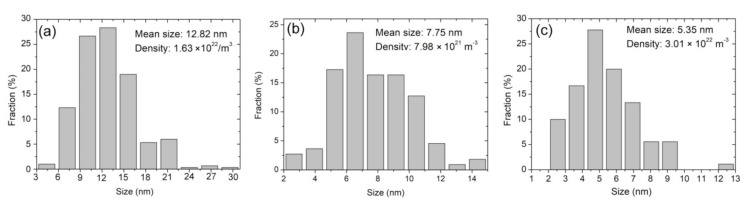
(**a**) Dislocation loop size and (**b**) cavity–diameter distributions in the Fe and He co-irradiated SIMP steel to fluences of 1.65 × 10^16^ Fe^3+^·cm^−2^ and 4.5 × 10^15^ He^+^·cm^−2^, (**c**) dislocation loop size distribution in the He implantation with Fe pre-irradiation into SIMP steel to fluences of 1 × 10^16^ Fe^10+^·cm^−2^ and 5 × 10^14^ He^+^·cm^−2^. It shows a skewed distribution in (**a**–**c**).

The size of the observed dislocation loops in Figure 2a was measured and the result is illustrated in Figure 5a. These dislocation loops have size ranging from 4.5–29 nm. The mean size is 12.82 nm and the number density is approximately 1.63 × 10^22^ m^−3^. Similarly, the size of the observed cavities in Figure 2b was measured and the result is shown in Figure 5b. The size of these cavities ranges from 2.65–14.35 nm. The mean size is 7.75 nm and the number density is approximately 7.98 × 10^21^ m^−3^. In comparison, only dislocation loops were observed in the He implantation with Fe pre-irradiation into SIMP steel, and the observed dislocation loops under the two-beam condition with ***g*** = (200) has a mean size of 5.35 nm and a number density of 3.01 × 10^22^ m^−3^. According to the present experimental results, there are two differences of the observed lattice defects between the Fe and He co-irradiated SIMP steel and the He implantation with Fe pre-irradiation into SIMP steel. First, cavities were observed clearly in the Fe and He co-irradiated SIMP steel, but not in the He implantation with Fe pre-irradiation into SIMP steel. Tanaka et al. [27] evaluated the synergistic effect via single, dual and triple ion beams consisting of Fe^3+^, Fe^3+^ + He^+^ or Fe^3+^ + He^+^ + H^+^ in Fe-Cr ferritic alloys, and found the increase in cavity nucleation via dual beam irradiation with He. Wakai et al. [28] argued that swelling in ferritic/martensitic steels is significantly enhanced by the synergistic effect of Fe irradiation- induced damage, hydrogen and helium atoms. Similarly, Hu et al. [29] reported synergistic effect of helium and hydrogen for bubble swelling in ferritic/martensitic steel, and also argued the increasing irradiation swelling of ferritic/martensitic steel after helium and hydrogen implantation. Marian et al. [30] regarded that the synergistic effect of hydrogen-helium depends on irradiation temperature. At lower temperatures (e.g., 470 °C and below), H and He atoms strongly interact with vacancies, but the mobility of these clusters is slow and there are many nano-scaled cavities. It is consistent with our present result that cavities with an average diameter of 8.4 nm were observed in the Fe and He co-irradiated sample. Secondly, the mean size of observed dislocation loops is larger, while the number density is lower after the Fe and He co-irradiation, compared to the He implantation with Fe pre-irradiation. This experimental phenomenon can be attributed to the increasing defect migration due to atomic collision. It is well known that energetic charged particles interact with both electrons and atoms in materials. Kinetic energy transfers to atoms can induce the atom displacement from their original sites, forming interstitial-type defects. The irradiation-induced disorder increases nonlinearly with irradiation dose [31]. With increasing defect concentration, more formed defects start recombination based on radiation-enhanced and diffusion defect reaction rate theory (e.g., high defect concentrations and modified energy barriers due to the formation of charge defects and charge redistribution) [31,32]. Radiation-enhanced diffusion induces the Ostwald ripening of oxide nanoparticles in oxide dispersion strengthened (ODS) steels after Fe irradiation [33]. In addition, the decrease in the threshold temperature for recrystallization during ion collision of amorphous SiC based on the model of ion-beam-induced random nucleation has been reported [34,35,36], similar phenomenon reported in Si [37]. In addition, the decrease in total lattice disorder after ions implantation or swift-heavy ions irradiation has been observed in disordered SiC [32,38,39,40,41,42]. The same mechanism can explain our experimental results that an obvious increase in the migration rate of lattice defects during Fe and He co-irradiation, resulting in the increase in the mean size, but the decrease in the number density of the observed dislocation loops. Meanwhile, the cavity formation depends on the concentration and size of vacancy-helium clusters. The increase in the migration rate of helium atoms and vacancies induces the formation of vacancy-helium clusters easily, and therefore cavities were observed in the region of helium atom deposition (see Figure 2b). On the contrary, initial Fe irradiation and then He implantation, Fe irradiation-induced defects migrate and accumulate to form clusters, such as vacancies, which are stable, and therefore these defects are hard to migrate during He implantation at a low fluence [32]. The microstructure in the Fe irradiation at 300 °C presents the same morphology with the Fe pre-irradiation + He implantation, as shown in Figure 4c. There are only dislocation loops in the damaged region. Therefore, the influence of He implantation on the Fe pre-irradiation-induced defects can be neglected. As a result, the mean size of the observed dislocation loops is smaller, while the number density of the observed dislocation loops is larger in the He implantation with Fe pre-irradiation into SIMP steel. More important, no cavities are observed in the helium deposition region. The hardness change among these three different irradiations also shows the pronounced irradiation hardening in the Fe and He co-irradiated sample compared to the others. The additional hardening should relate to helium cavities in the damaged layer. It is consistent with report of Ando et al. [43] that extra radiation hardening in the Fe and He co-irradiated F82H due to cavities in the damaged layer. Compared with the co-irradiation, He injection via the subsequent irradiation cannot give effective hardening.

It should be noted that radiation-induced precipitation in nuclear structural materials has been widely reported. Tunes et al. [44] recently reported 30 keV Xe-irradiated an austenitic stainless steel at 800 °C and argued that inert gas bubbles can accelerate clustering and precipitation kinetics. Similarly, vacancy-type defects can enhance the nucleation of carbide precipitations in Ar-irradiated AISI 316L alloys [45]. Fang et al. [46] investigated Fe-irradiated SIMP steel at 300 °C and 400 °C and regarded the Si element enrichment and Ta element depletion inside the precipitates after 1 dpa irradiation at 300 °C. In addition, thermodynamics of the SIMP steel after irradiation should be concerned. Tunes et al. [47] observed α′ and M_23_C_6_ precipitates formed in the 30 keV Xe irradiated AISI-348 steel at 800 °C. A equilibrium state can be reached by ion irradiation. Radiation-induced precipitation and radiation-assisted thermodynamic equilibrium can be accounted for the increase in diffusion rate of solute atoms by the exchange of atoms with point defects. In this study, the selected zone diffraction patterns do not present extra diffraction spots, except for matrix, indicating no precipitation formed after irradiation. However, the resolution of the selected zone diffraction pattern is limited. In particular, the growth of carbide precipitates is slow at 300 °C irradiation [48] and, therefore, these new phases have weak diffraction spots as compared to matrix. To confirm irradiation-induced precipitation in SIMP steel, it needs scanning transmission electron microscopy and energy dispersive X-ray spectroscopy to analyze element distribution in the damaged zone.

**Figure 6:**
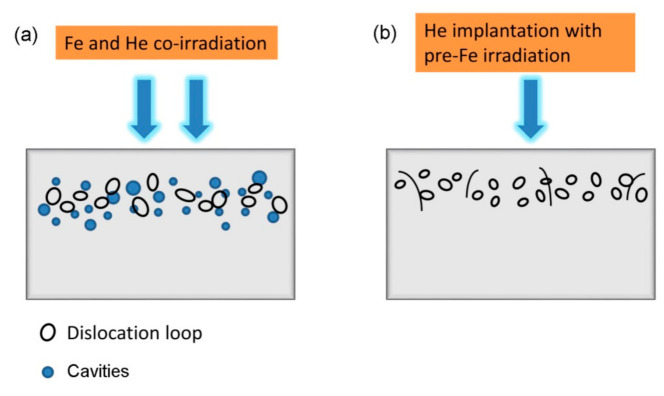
A schematic diagram showing microstructural defects in the Fe and He co-irradiated SIMP steel (**a**) or He implantation with Fe pre-irradiation into SIMP steel at 300 °C (**b**).

A schematic diagram presented in Figure 6 illustrates the lattice disorder observed in the two irradiation conditions. There are cavities and dislocation loops formed in the Fe and He co-irradiated SIMP steel at 300 °C, while only dislocation loops were formed in the He implantation with Fe pre-irradiation into SIMP steel. Similarly, no cavities, but only dislocation loops were observed in the Fe irradiated SIMP, as illustrated in Figure 6.

## 4. Conclusions

In this study, a comparison between Fe and He co-implantation and He implantation with Fe pre-irradiation of SIMP steel has been investigated by nano-indenter and XTEM. Irradiation hardening was found more pronounced in the simultaneously co-irradiated samples compared to the subsequently irradiated sample. Almost identical hardness profiles were obtained for the Fe irradiated sample and for the He implanted sample with Fe pre-irradiation. Lattice defects, including dislocation loops and cavities were observed by TEM. By analyzing the observed lattice defects, synergistic effect of simultaneous Fe and He co-irradiation have been observed at 300 °C irradiation, where cavities with a mean size of 7.75 nm are randomly distributed. Moreover, the growth of dislocation loops with a mean size of 12.82 nm was found in the damaged layer, compared to no cavities but small dislocation loops in the subsequent irradiation. Based on the present study, we conclude that subsequent irradiation does not provide an adequate replacement for dual-beam or triple-beam irradiation.

## Figures and Tables

**Table 1:** Nominal composition of SIMP steel (wt.%).

Steels	Fe	Cr	Si	W	Mn	C	V	Nb	P
SIMP	Bal	10.7	1.4	1.2	0.5	0.2	0.2	0.01	0.004

## Data Availability

The data presented in this study are available on request from the corresponding author.
